# Influence of beetroot juice supplementation on intermittent exercise performance

**DOI:** 10.1007/s00421-015-3296-4

**Published:** 2015-11-27

**Authors:** Lee J. Wylie, Stephen J. Bailey, James Kelly, James R. Blackwell, Anni Vanhatalo, Andrew M. Jones

**Affiliations:** Sport and Health Sciences, College of Life and Environmental Sciences, St. Luke’s Campus, University of Exeter, Heavitree Road, Exeter, Devon EX1 2LU UK

**Keywords:** Nitric oxide, Beetroot juice, Repeated sprint exercise, Exercise performance, Team sports

## Abstract

**Purpose:**

This study tested the hypothesis that nitrate (NO_3_^−^) supplementation would improve performance during high-intensity intermittent exercise featuring different work and recovery intervals.

**Method:**

Ten male team-sport players completed high-intensity intermittent cycling tests during separate 5-day supplementation periods with NO_3_^−^-rich beetroot juice (BR; 8.2 mmol NO_3_^−^ day^−1^) and NO_3_^−^-depleted beetroot juice (PL; 0.08 mmol NO_3_^−^ day^−1^). Subjects completed: twenty-four 6-s all-out sprints interspersed with 24 s of recovery (24 × 6-s); seven 30-s all-out sprints interspersed with 240 s of recovery (7 × 30-s); and six 60-s self-paced maximal efforts interspersed with 60 s of recovery (6 × 60-s); on days 3, 4, and 5 of supplementation, respectively.

**Result:**

Plasma [NO_2_^−^] was 237 % greater in the BR trials. Mean power output was significantly greater with BR relative to PL in the 24 × 6-s protocol (568 ± 136 vs. 539 ± 136 W; *P* < 0.05), but not during the 7 × 30-s (558 ± 95 vs. 562 ± 94 W) or 6 × 60-s (374 ± 57 vs. 375 ± 59 W) protocols (*P* > 0.05). The increase in blood [lactate] across the 24 × 6-s and 7 × 30-s protocols was greater with BR (*P* < 0.05), but was not different in the 6 × 60-s protocol (*P* > 0.05).

**Conclusion:**

BR might be ergogenic during repeated bouts of short-duration maximal-intensity exercise interspersed with short recovery periods, but not necessarily during longer duration intervals or when a longer recovery duration is applied. These findings suggest that BR might have implications for performance enhancement during some types of intermittent exercise.

## Introduction

Nitric oxide (NO) is a multi-functional physiological signaling molecule that can be endogenously derived from the oxygen (O_2_)-dependent catabolism of l-arginine in a reaction catalyzed by the NO synthase (NOS) enzymes (Stamler and Meissner 2001), or from the O_2_-independent reduction of nitrite (NO_2_^−^) by numerous NO_2_^−^ reductases (Lundberg and Weitzberg 2009). In recreationally active and moderately trained subjects, increasing the circulating plasma [NO_2_^−^] via inorganic nitrate (NO_3_^−^) supplementation has been reported to improve performance during sub-maximal endurance exercise in most (e.g., Bailey et al. 2009; Cermak et al. 2012; Porcelli et al. 2015; Wylie et al. 2013a; see Jones 2014 for review), but not all (e.g., Kelly et al. 2014), previous studies. However, the reduction of NO_2_^−^ to NO, and therefore the potential for NO-mediated physiological signaling following NO_3_^−^ supplementation, is potentiated as O_2_ tension (Castello et al. 2006) and pH (Modin et al. 2001) decline. Given that muscle PO_2_ and pH decline to a greater extent (Richardson et al. 1995) at higher exercise intensities, NO_3_^−^ supplementation may be more likely to increase NO synthesis, and perhaps improve performance, at higher exercise intensities.

Fatigue development during high-intensity intermittent exercise is linked, in part, to the decline in muscle phosphocreatine concentration [PCr] (Fulford et al. 2013; Gaitanos et al. 1993), whereas recovery of intermittent exercise performance is linked to muscle PCr resynthesis (Bogdanis et al. 1995, 1996; Mendez-Villanueva et al. 2012). Importantly, NO_3_^−^ supplementation has been shown to lower the PCr cost of force production during high-intensity intermittent exercise (Fulford et al. 2013), which might delay the attainment of a critically low muscle [PCr] during intermittent exercise (Chidnok et al. 2013). Moreover, the increased perfusion and oxygenation of type II muscle that has been reported following NO_3_^−^ supplementation (Ferguson et al. 2013, 2015) might facilitate the O_2_-dependent recovery of PCr (Trump et al. 1996; Vanhatalo et al. 2011) in the type II muscle fibers that are preferentially recruited during high-intensity intermittent exercise (Essén 1978; Green 1978; Krustrup et al. 2004, 2009; Thomson et al. 1979). Supplementation with NO_3_^−^ has also been reported to: enhance calcium handling and augment the rate of force development in type II muscle fibers (Hernández et al. 2012); and to increase force production (Coggan et al. 2015; Haider and Folland 2014) and attenuate fatigue development at high muscle contraction frequencies (Bailey et al. 2015) and when the proportional contribution of type II muscle to force production is expected to be increased (Breese et al. 2013). These findings suggest that NO_3_^−^ supplementation might improve performance during high-intensity intermittent exercise. Moreover, given that the recovery of muscle PCr and force production during intermittent exercise is incomplete when shorter recovery durations are applied (Bogdanis et al. 1995), it is possible that NO_3_^−^ supplementation might be most effective during high-intensity intermittent exercise when the recovery duration between work intervals is relatively short.

Given that NO_3_^−^ supplementation has been shown to improve (Aucouturier et al. 2015; Bond et al. 2012; Thompson et al. 2015; Wylie et al. 2013a), compromise (Martin et al. 2014) or have no effect (Christensen et al. 2013; Muggeridge et al. 2013) on high-intensity intermittent exercise performance, the potential for this supplement to be ergogenic in intermittent exercise is controversial. These equivocal findings might be attributed to inter-study differences in the training status of the participants, the exercise modality employed, the NO_3_^−^ supplementation procedures, and differences in the intermittent exercise protocols (work and rest intensities, work and rest durations, work-to-rest ratio, and number of work intervals). Further research is required to elucidate the relative efficacy of NO_3_^−^ supplementation in enhancing performance in different intermittent exercise performance protocols.

The purpose of this investigation was to assess the effects of NO_3_^−^ supplementation on performance during a variety of high-intensity intermittent exercise tests using the same subject population, exercise modality, and NO_3_^−^ dosing procedures. We hypothesized that: (1) NO_3_^−^ supplementation would improve performance during all high-intensity intermittent protocols administered and (2) performance would be enhanced to the greatest extent when maximal-intensity intermittent exercise was accompanied by the shortest recovery duration.

## Methods

### Subjects

Ten male recreational team-sport players (mean ± SD: age 21 ± 1 years, body mass 87.5 ± 9.5 kg, height 1.82 ± 0.01 m, $\dot{V}$O_2peak_, 58 ± 8 mL kg^−1^ min^−1^) familiar with intense intermittent exercise volunteered to participate in this study. Prior to testing, subjects were informed of the protocol and the possible risks and benefits of participation before written informed consent was obtained. All procedures were approved by the Institutional Ethics Committee and conformed to the code of ethics of the Declaration of Helsinki.

### Experimental design

The subjects reported to the laboratory on eight separate occasions over a ~4-week period. During the first visit to the laboratory, subjects initially performed a ramp incremental test for assessment of ramp test peak power and peak $\dot{V}$O_2_ ($\dot{V}$O_2peak_). After 30 min of passive recovery, subjects completed 5 × 6-s all-out cycle sprints interspersed by 24 s of recovery and, following an additional 10 min of passive recovery, 2 × 30-s all-out cycle efforts interspersed by 4 min of recovery. After a minimum of 24 h recovery, subjects returned to the laboratory to complete 6 × 60-s self-paced maximal efforts separated by 60 s of active recovery. These sprints and self-paced efforts served as a familiarization to the three experimental protocols that are outlined in detail below.

Following completion of the preliminary testing, subjects were assigned in a randomised, double-blind, cross-over experimental design to receive either NO_3_^−^-rich beetroot juice (BR) or NO_3_^−^-depleted beetroot juice (PL) for 5 days. Subjects completed 24 × 6-s all-out sprints, 7 × 30-s all-out sprints, and 6 × 60-s self-paced maximal efforts on days 3, 4, and 5 of supplementation, respectively. A washout period of at least 7 days separated each supplementation period. Subjects were asked to record their food intake in the 24 h preceding the first experimental trial and to replicate this same diet in the 24 h preceding all subsequent trials. Subjects were instructed to arrive at the laboratory in a rested and fully hydrated state, at least 3 h post-prandial, and to avoid strenuous exercise in the 24 h preceding each testing session. Subjects were also asked to refrain from caffeine and alcohol intake 6 and 24 h before each laboratory visit, respectively, and to abstain from antibacterial mouthwash and chewing gum use throughout the study as these products blunt the reduction of nitrate to nitrite in the oral cavity (Govoni et al. 2008). All tests were performed at the same time of day (±1 h).

### Determination of peak oxygen uptake and ramp test peak power

All exercise tests were performed on an electrically braked cycle ergometer (Lode Excalibur Sport, Groningen, The Netherlands). The ergometer saddle and handle bar height configuration was recorded and reproduced in all subsequent tests. The ramp incremental test protocol consisted of 3 min of “unloaded” baseline cycling at 20 W followed by a linear 30 W min^−1^ increase in power output until volitional exhaustion. Subjects were instructed to maintain a cadence of 80 rpm and, when cadence fell below 70 rpm despite strong verbal encouragement, the test was terminated and peak power was recorded. Pulmonary gas exchange was measured throughout the test on a breath-by-breath basis and subsequently averaged into 10-s bins for analysis. $\dot{V}$O_2peak_ was calculated as the mean value over the final 30 s of exercise.

### Intermittent exercise tests

Before the onset of all intermittent exercise tests, subjects completed a standardized warm-up that comprised 5 min of cycling at 80 W (80 rpm), followed immediately by 3 × 3-s sprints, each interspersed by 20 s of passive recovery. Five minutes after completing the warm-up protocol, subjects performed one of the individual intermittent protocols as described below.

#### 24 × 6-s protocol

After undergoing the standardized warm-up, subjects completed a single 6-s all-out sprint. The mean power output (MPO) recorded in this 6-s sprint was used as a criterion score during the subsequent 24 × 6-s cycle test. Upon completion of this sprint, subjects rested for 5 min before completing the 24 × 6-s test, which consisted of 24 6-s all-out sprints departing every 30 s. To mitigate the potential confounding influence of different pacing strategies between supplements (Waldron and Highton 2014), the MPO during sprint 1 of the 24 × 6-s protocol was required to equal or exceed 95 % of the MPO during the benchmark sprint (Fitzsimons et al. 1993). Participants met this criterion in all tests. All 6-s sprints were performed in a standing position and interspersed by 24 s of passive seated recovery.

#### 7 × 30-s protocol

The 7 × 30-s protocol consisted of seven 30-s all-out cycle sprints interspersed with 4 min of recovery. Each 4 min recovery period consisted of 210 s of active recovery at 20 W followed by 30 s of passive recovery. Subjects remained seated during all 30-s sprints and recovery periods. During all 6- and 30-s sprints, subjects were verbally encouraged to perform with maximum effort, but were not informed of the sprint number to prevent pacing.

#### 6 × 60-s self-paced protocol

The 6 × 60-s protocol consisted of six 60-s self-paced cycle efforts interspersed with 60 s of recovery. Subjects were instructed to achieve the highest MPO across all 60-s efforts. Each 60-s recovery period consisted of 40 s of active recovery at 20 W (‘unloaded’) and 20 s of seated passive recovery. Subjects remained seated during all 60-s self-paced work intervals and recovery periods. Subjects were not provided with any verbal encouragement or information pertaining to the interval number until the final 60-s effort. At this point, subjects were instructed to maximize MPO.

Five seconds before the onset of each work interval, subjects were asked to position the right crank 45° down from the vertical axis. Subsequently, all work intervals were preceded by a 3-s countdown followed by a clear “GO” command. The resistance during each sprint and self-paced exercise bout was applied using the cadence-dependent linear function (linear factor = power/cadence squared) of the Lode ergometer. The fixed resistance for the 6- and 30-s sprints was set so that upon attaining a cadence of 120 rpm, subjects would achieve a power output equivalent to 270 and 220 % of their ramp test peak power, respectively. These resistances were selected, based on findings from pilot experiments, as they allowed subjects to achieve peak power output (PPO) at ~120 rpm, which is the optimal cadence for attainment of PPO during all-out cycling exercise (McCartney et al. 1985). The fixed resistance during the 60-s self-paced exercise bouts was set so that subjects would achieve 80 % of their ramp peak power at 80 rpm. Subjects were blinded to the elapsed exercise time in the 24 × 6-s and 7 × 30-s intermittent exercise performance tests.

### Supplementation

Following completion of the pre-supplementation tests, subjects were assigned using a randomised, balanced, cross-over design to receive concentrated NO_3_^−^-rich beetroot juice (BR; containing ~4.1 mmol of NO_3_^−^ per 70 mL; Beet It, James White Drinks Ltd., Ipswich, UK) and NO_3_^−^-depleted beetroot juice (PL; containing ~0.04 mmol NO_3_^−^ per 70 mL; Beet It, James White Drinks Ltd., Ipswich, UK) for 5 days. On non-experimental days (days 1 and 2) of each supplementation period, subjects consumed 1 × 70 mL in the morning (~10 a.m.) and 1 × 70 mL in the evening (~7 p.m.). On experimental days (days 3, 4, and 5 of supplementation), subjects consumed 2 × 70 mL 2.5 h prior to the onset of testing procedures. This dose of BR and the timing of ingestion was selected based on our previous research which suggested that 8.4 mmol of NO_3_^−^ (administered as 140 mL BR) resulted in a peak increase in plasma [nitrite] 2–3 h later that coincided with improved exercise tolerance (Wylie et al. 2013a). On experimental days 1 and 2 for both BR and PL (days 3 and 4 of supplementation), subjects consumed a further 1 × 70 mL dose 3 h post-completion of testing procedures.

### Measurements and data analysis procedures

#### Performance variables

Power output was recorded continuously at 1 Hz throughout each exercise test using customized software (created through Labview) and exported for subsequent analysis. For the 24 × 6-s and 7 × 30-s tests, MPO and PPO were calculated for each sprint. In addition, the mean of MPO (MPO_mean_) was calculated for each protocol and mean of PPO (PPO_mean_) was calculated for the 24 × 6-s and 7 × 30-s protocols. To assess performance at different stages of the 24 × 6-s protocol, MPO and PPO data were pooled into bins of six sprints (i.e. sprints 1–6, 7–12, 13–18, 19–24) prior to statistical analysis.

#### Pulmonary gas exchange and ventilation

Pulmonary gas exchange and ventilation were collected breath-by-breath in all exercise tests. Subjects wore a nose clip and breathed through a low-dead space (90 mL), low-resistance (0.75 mmHg L^−1^ s^−1^ at 15 L/s^−1^) mouthpiece and impeller turbine assembly (Jaeger Triple V). The inspired and expired gas concentration signals were continuously sampled using paramagnetic (O_2_) and infrared (CO_2_) analyzers (Oxycon Pro; Jaeger, Hoechberg, Germany) via a capillary line connected to the mouthpiece. These analyzers were calibrated before each test with gases of known concentration, and the turbine volume transducer was calibrated using a 3-L syringe (Hans Rudolph, Kansas City, MO). Breath-by-breath $\dot{V}$O_2_ data from each test were linearly interpolated to provide second-by-second values. Subsequently, mean $\dot{V}$O_2_ was assessed during each work and recovery period and averaged to provide the overall mean $\dot{V}$O_2_ during the work and recovery periods for each intermittent exercise test. The mean $\dot{V}$O_2_ across all interval and recovery periods for each intermittent exercise test was also calculated.

#### Venous and capillary blood sampling

Upon arrival at the laboratory for each intermittent exercise test, a venous blood sample was taken for the determination of plasma [NO_2_^−^]. Venous blood samples were drawn into 7.5 mL lithium–heparin tubes (Monovette lithium heparin; Sarstedt, Leicester, UK). Within 1 min of collection, samples were centrifuged at 4000 rpm and 4 °C for 7 min. Plasma was subsequently aliquoted and immediately frozen at −80 °C for later analysis of [NO_2_^−^] as described previously (Wylie et al. 2013a, b).

Capillary blood samples were collected from a fingertip into a capillary tube prior to the warm-up procedure and 20 s prior to the onset of each exercise test. Additionally, capillary blood samples were collected after every two sprints in the 24 × 6-s protocol and after every exercise interval in the 7 × 30-s and 6 × 60-s protocols. These samples were stored on ice and analyzed within 5 min of collection to determine blood lactate concentration [lactate] using an automated blood [lactate] analyzer (YSI 1500; Yellow Springs Instrument, Yellow Springs, OH).

### Statistical analysis

Between-supplement differences in MPO_mean_, PPO_mean_, and the overall changes in pulmonary $\dot{V}$O_2_ were analyzed using a paired-sample *t* test for each intermittent performance test. Changes in plasma [NO_2_^−^] recorded on days 3, 4, and 5 of supplementation were determined via a two-way (supplement × test) repeated-measures ANOVA. Likewise, alterations in power output, blood [lactate], and pulmonary $\dot{V}$O_2_ for work and recovery intervals in the 24 × 6-s, 7 × 30-s and 6 × 60-s protocols were assessed via two-way (supplement × interval) repeated-measures ANOVAs. Significant effects were further explored using Fisher’s LSD. Statistical significance was accepted at *P* < 0.05. Results are presented as mean ± SD unless otherwise stated.

## Results

Subjects reported that they consumed all servings of each supplement at the required times and that their diet and exercise habits prior to each experimental test were consistent.

### Plasma [NO_2_^−^]

**Fig. 1:**
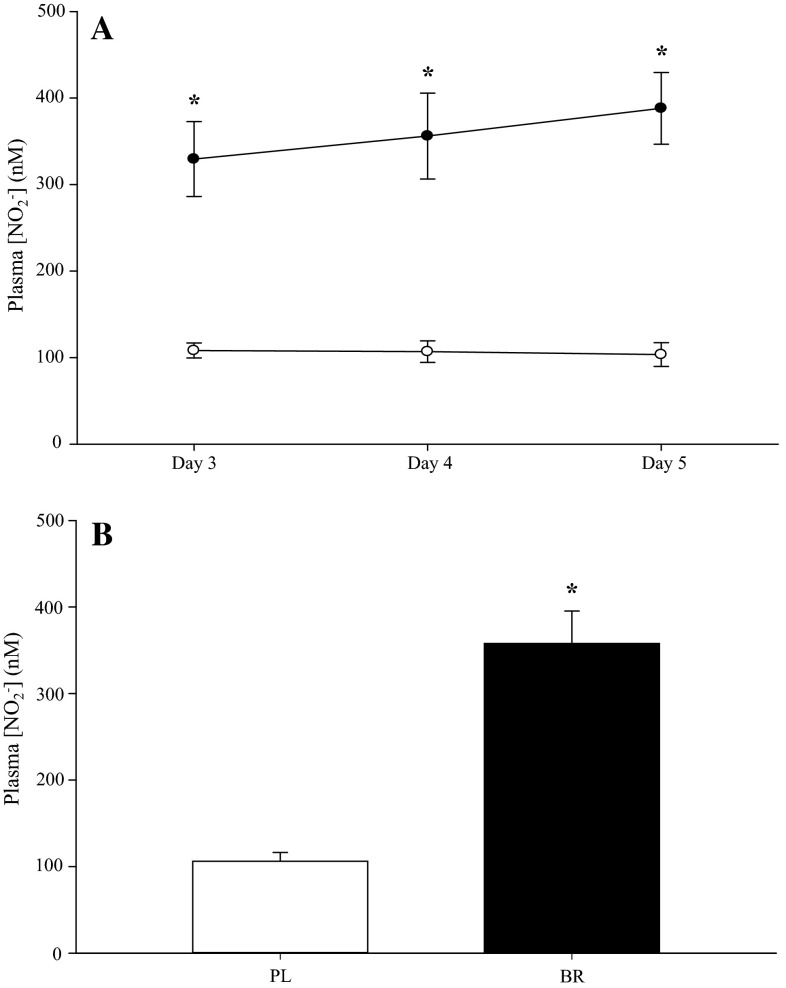
Plasma nitrite concentration ([NO_2_^−^]) was elevated in BR (*closed circles*) compared to PL (*open circles*) on days 3, 4 and 5 of supplementation (**a**). On average across the three sample points, plasma [NO_2_^−^] was 237 % higher in BR (*filled bar*) compared to PL (*open bar*) (**b**). *Error bar* indicates the SE. *P < 0.05 compared to PL

The group mean plasma [NO_2_^−^] values obtained on days 3, 4, and 5 of the BR and PL supplementation periods are shown in Fig. 1. Plasma [NO_2_^−^] was elevated during the BR supplementation compared to PL at all sample points (*P* < 0.05; Fig. 1a). The mean increase in plasma [NO_2_^−^] with BR ingestion across the three sample points was 237 % (BR 358 ± 119 vs. PL 106 ± 32 nM; *P* < 0.05; Fig. 1b). Plasma [NO_2_^−^] was not significantly different across days 3–5 in PL or BR (*P* > 0.05; Fig. 1a).

### Power output

**Fig. 2:**
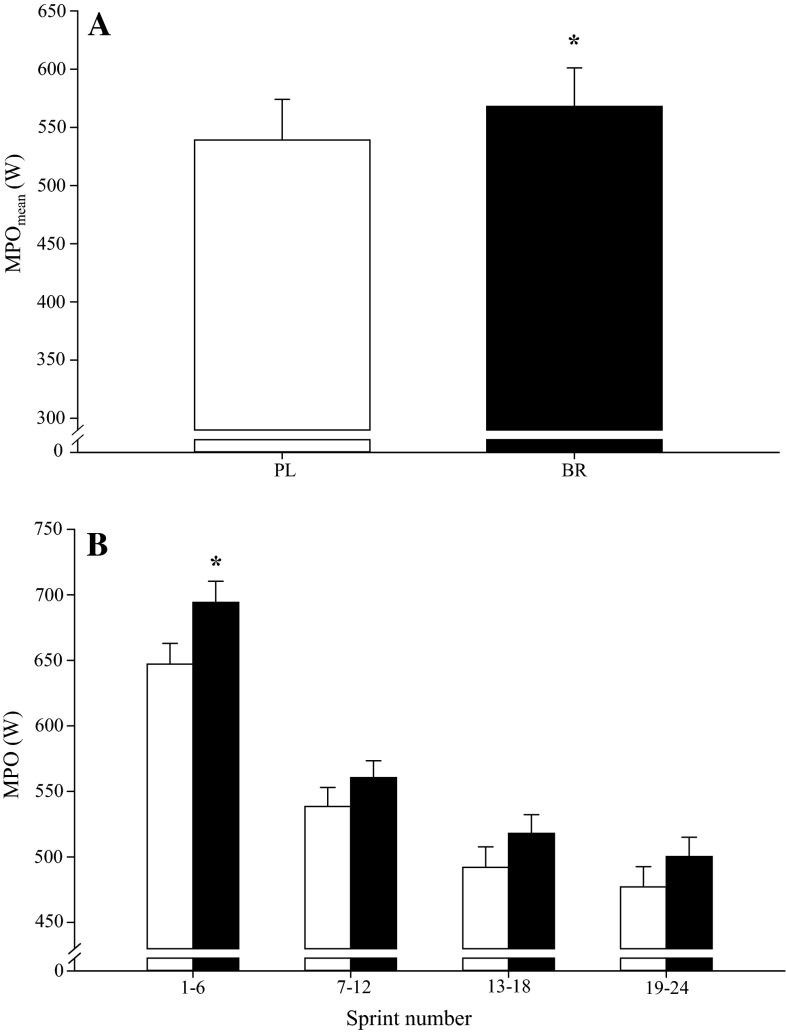
The mean power output (MPO_mean_) across the 24 × 6-s sprint protocol was 5 % greater with BR (*filled bars*) relative to PL (*open bars*) (**a**). Specifically, mean power output (MPO) was greater with BR in sprints 1–6, but not in sprints 7–12, 13–18 or 19–24, when compared to PL (**b**). *Error bars* indicate the SE. *P < 0.05 compared to PL

**Fig. 3:**
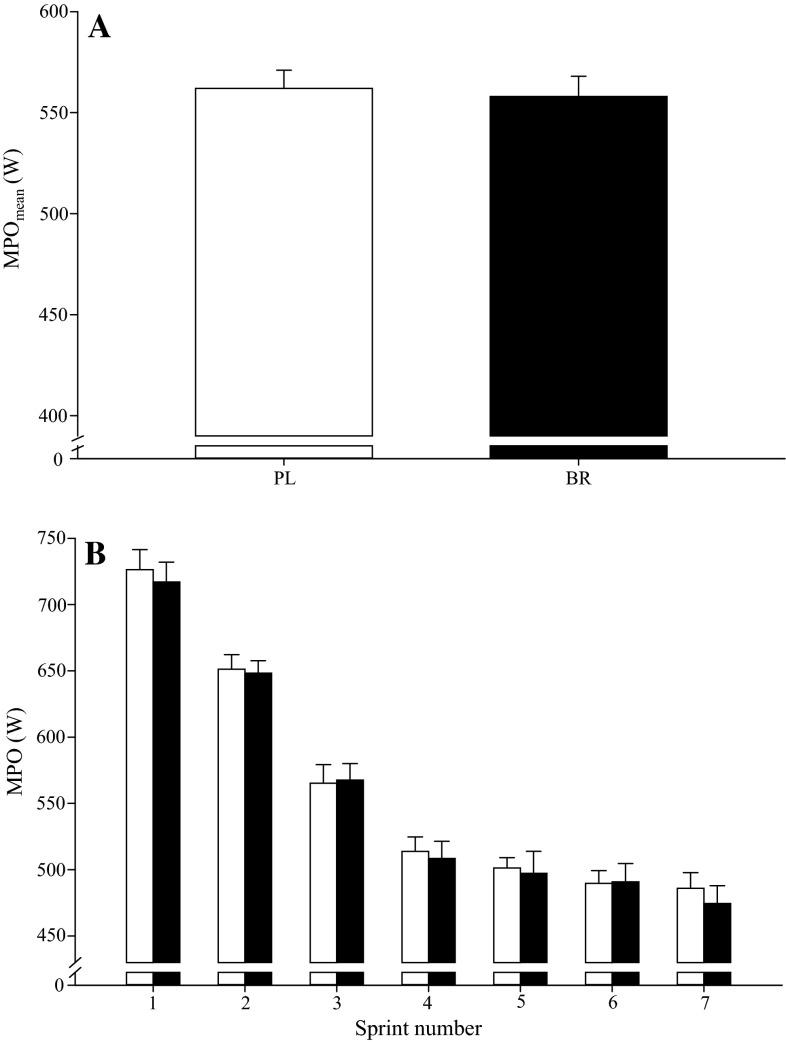
The mean power output (MPO_mean_) across the 7 × 30-s protocol (**a**) and mean power output (MPO) during each individual sprint (**b**) were not different between BR (*filled bars*) and PL (*open bars*). *Error bars* indicate the SE

**Fig. 4:**
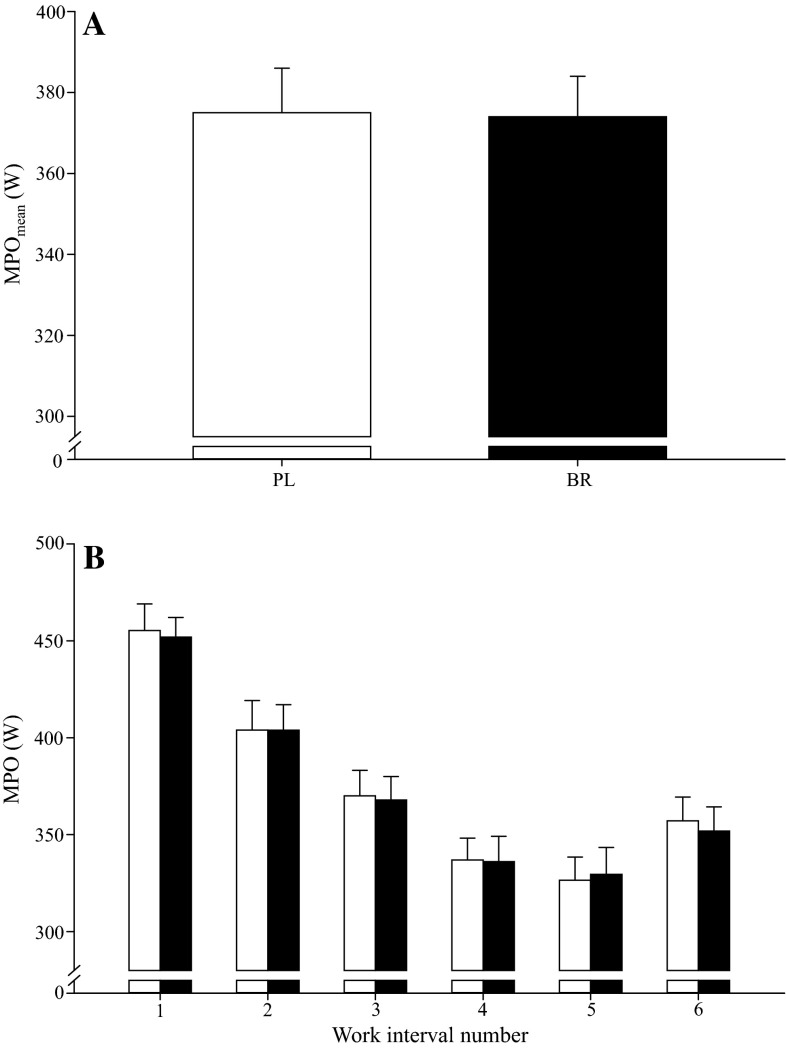
The mean power output (MPO_mean_) across the 6 × 60-s protocol (**a**) and mean power output (MPO) during each individual exercise interval (**b**) were not different between BR (*filled bars*) and PL (*open bars*). *Error bars* indicate the SE

The power output data during the 24 × 6-s, 7 × 30-s and 6 × 60-s protocols are illustrated in Figs. 2, 3, and 4, respectively.

#### 24 × 6-s protocol

There was no significant difference in PPO_mean_ between BR (792 ± 159 W) and PL (782 ± 154 W; *P* > 0.05). However, compared to PL, MPO_mean_ was significantly greater following BR supplementation (BR 568 ± 136 vs. PL 539 ± 136 W; *P* < 0.05; Fig. 2a). Further analyses revealed that BR supplementation did not significantly increase MPO in any individual sprints (*P* > 0.05). However, when the 24 × 6-s sprints were pooled into four groups of six sprints, MPO was greater with BR in sprints 1–6 (BR 694 ± 125 vs. PL 647 ± 122 W; *P* < 0.05), but not in sprints 7–12 (BR 560 ± 100 vs. PL 539 ± 112 W; *P* > 0.05), 13–18 (BR 518 ± 111 vs. PL 492 ± 121 W; *P* > 0.05), and 19–24 (BR 500 ± 114 vs. PL 477 ± 119 W; *P* > 0.05; Fig. 2b).

#### 7 × 30-s protocol

There were no significant differences between BR and PL in PPO_mean_ (BR 768 ± 157 vs. PL 776 ± 142 W; *P* > 0.05) or MPO_mean_ (BR 558 ± 95 vs. PL 562 ± 94 W; *P* > 0.05; Fig. 3a) during the 7 × 30-s sprint exercise tests. There were also no differences between PL and BR in MP (Fig. 3b) and PP across individual sprints (*P* > 0.05).

#### 6 × 60-s protocol

The MPO_mean_ was not significantly different between PL (375 ± 59 W) and BR (374 ± 57 W; *P* > 0.05; Fig. 4a) in the 6 × 60-s exercise test. There were no differences in MP between PL and BR for any of the individual intervals (*P* > 0.05; Fig. 4b).

### Pulmonary gas exchange

Pulmonary gas exchange data collected during the three intermittent performance tests in BR and PL are displayed in Table 1. While pulmonary $\dot{V}$O_2_ and $\dot{V}$CO_2_ increased with time across the three intermittent exercise protocols (*P* < 0.05), mean pulmonary $\dot{V}$O_2_ and $\dot{V}$CO_2_ was not different between PL and BR during the work intervals, recovery periods or across the overall protocol in any of the exercise test protocols (all *P* > 0.05; Table 1). RER was not different between PL and BR during the work intervals, recovery periods or across the overall protocol in the 7 × 30-s and 6 × 60-s protocols (all *P* > 0.05; Table 1). However, compared to PL, RER was increased during the work intervals, recovery periods, and across the overall protocol in the 24 × 6-s protocol (all *P* < 0.05; Table 1).

**Table 1:** Mean (±SD) pulmonary gas exchange variables during the work intervals, recovery periods, and across the overall protocol in all three exercise test protocols following PL and BR supplementation

	24 × 6-s protocol		7 × 30-s protocol		6 × 60-s protocol	
	PL	BR	PL	BR	PL	BR
$\dot{V}$O_2_						
Work interval (L min^−1^)	3.66 ± 0.29	3.64 ± 0.31	2.57 ± 0.22	2.55 ± 0.26	3.33 ± 0.23	3.27 ± 0.32
Recovery period (L min^−1^)	3.11 ± 0.24	3.12 ± 0.28	1.64 ± 0.15	1.66 ± 0.16	2.73 ± 0.17	2.70 ± 0.28
Overall (L min^−1^)	3.22 ± 0.24	3.23 ± 0.28	1.74 ± 0.15	1.76 ± 0.16	3.02 ± 0.18	2.99 ± 0.29
$\dot{V}$CO_2_						
Work interval (L min^−1^)	4.11 ± 0.32	4.27 ± 0.41	2.50 ± 0.23	2.49 ± 0.30	3.61 ± 0.31	3.57 ± 0.36
Recovery period (L min^−1^)	3.39 ± 0.28	3.52 ± 0.32	2.00 ± 0.16	2.02 ± 0.18	3.50 ± 0.27	3.50 ± 0.28
Overall (L min^−1^)	3.53 ± 0.28	3.67 ± 0.33	2.05 ± 0.16	2.07 ± 0.18	3.55 ± 0.27	3.53 ± 0.32
RER						
Work interval	1.13 ± 0.01	1.18 ± 0.04*	1.04 ± 0.04	1.04 ± 0.06	1.12 ± 0.03	1.13 ± 0.03
Recovery period	1.09 ± 0.02	1.13 ± 0.05*	1.26 ± 0.06	1.26 ± 0.04	1.33 ± 0.10	1.35 ± 0.10
Overall	1.10 ± 0.02	1.14 ± 0.05*	1.24 ± 0.05	1.23 ± 0.04	1.23 ± 0.06	1.24 ± 0.06

* Significantly different from PL (*P* < 0.05)

### Blood [lactate]

**Fig. 5:**
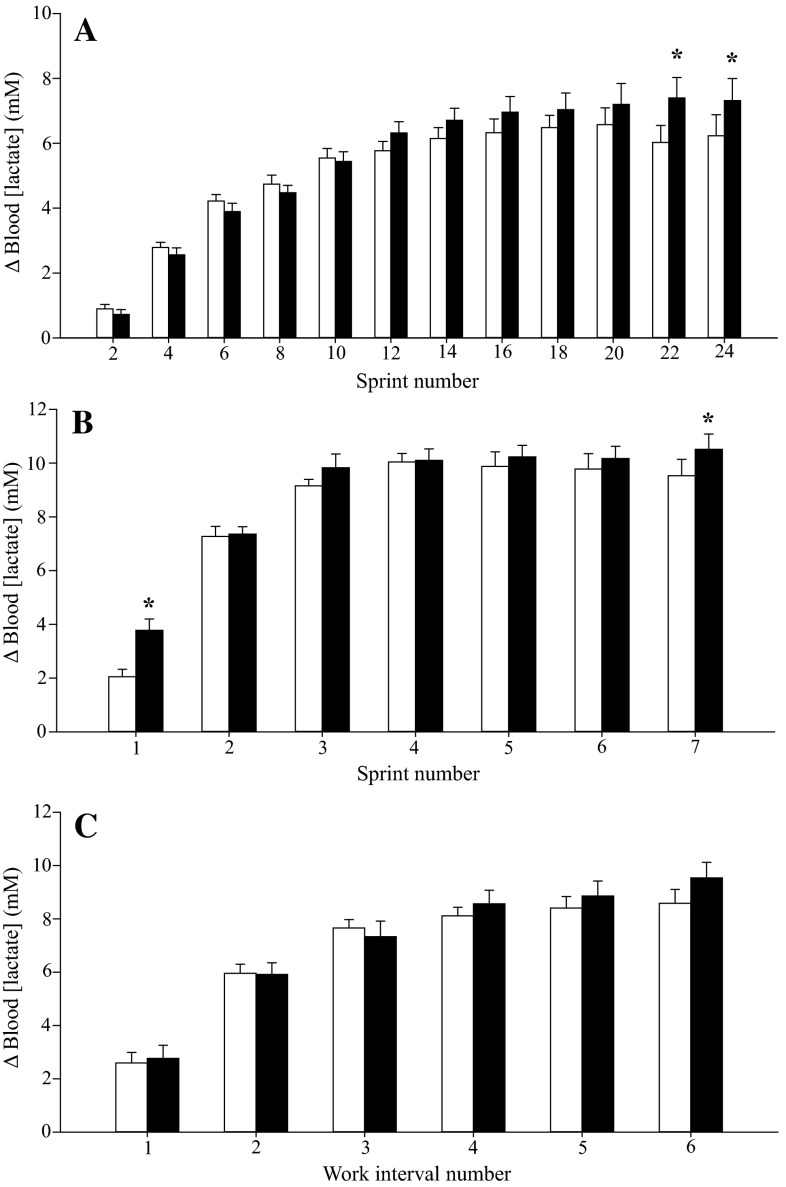
Change (**∆)** relative to pre-exercise baseline in blood lactate concentration ([lactate]) during the 24 × 6-s (**a**), 7 × 30-s (**b**) and 6 × 60-s (**c**) exercise protocols, following PL (*open bars*) and BR (*filled bars*) supplementation. Note the greater **∆** in blood [lactate] with BR after sprints 22 and 24 in the 24 × 6-s protocol, and after sprints 1 and 7 in the 7 × 30-s protocol. *Error bars* indicate the SE. *P < 0.05 compared to PL

Blood [lactate] was not significantly different between BR and PL at baseline (before completion of the warm-up procedure) in any test protocol (all *P* > 0.05). The change in blood [lactate] during the intermittent exercise tests with BR and PL is shown in Fig. 5.

#### 24 × 6-s protocol

The change in blood [lactate] from pre-exercise to the completion of sprint 24 was significantly greater in BR compared to PL (BR 7.3 ± 2.2 vs. PL 6.2 ± 2.1 mM; *P* < 0.05; Fig. 5a). The rise in blood [lactate] above the pre-exercise value was also significantly greater with BR after sprint 22 (*P* < 0.05), but not in the earlier sprints (*P* > 0.05; Fig. 5a).

#### 7 × 30-s protocol

The change in blood [lactate], from pre-exercise to the completion of sprint 7, was significantly greater in BR compared to PL (BR 10.5 ± 1.8 vs. PL 9.5 ± 1.9 mM; *P* < 0.05; Fig. 5b). BR supplementation also resulted in a significantly greater ∆ blood [lactate] post-sprint 1 (BR 3.8 ± 1.3 vs. PL 2.0 ± 0.9 mM; *P* < 0.05), but not post-sprints 2–6 (*P* > 0.05; Fig. 5b).

#### 6 × 60-s protocol

The change in blood [lactate], from baseline, to post completion of interval 6, was not significantly different between supplements (BR 9.5 ± 1.8 vs. PL 8.6 ± 1.6 mM; *P* = 0.07; Fig. 5c). Furthermore, ∆ blood [lactate] was not significantly impacted by BR post-intervals 1–5 (*P* > 0.05; Fig. 5c).

## Discussion

The principal original finding of this study was that short-term supplementation with BR significantly improved mean power output during 24 6-s all-out sprints interspersed with 24 s of recovery but not during protocols comprising seven 30-s all-out sprint efforts interspersed with 4 min of recovery or six 60-s self-paced maximal efforts interspersed with 60 s of recovery. BR was especially effective in improving MPO_mean_ in the early part of the 24 × 6-s protocol, with the difference between conditions being significantly different in the first 6 but not the subsequent 18 sprints. These findings suggest that BR supplementation may improve performance during high-intensity intermittent exercise when short-duration, maximal-intensity intervals are repeated with a short recovery duration, but not when interval and recovery durations are longer.

Consistent with previous studies, the circulating plasma [NO_2_^−^] was significantly increased following the short-term BR supplementation regimen employed in this study (Bailey et al. 2009, 2010, 2015; Breese et al. 2013; Thompson et al. 2015; Vanhatalo et al. 2011; Wylie et al. 2012, 2013a, b). Importantly, plasma [NO_2_^−^] was elevated above the corresponding PL trials by a similar magnitude on each of the BR trials. Therefore, the relative efficacy of BR to improve performance in the intermittent tests cannot be attributed to differences in plasma [NO_2_^−^] between tests. An increase in plasma [NO_2_^−^] represents an increased ‘substrate’ for NO synthesis through the O_2_-independent reduction of NO_2_^−^ to NO (Lundberg and Weitzberg 2009). A comparable BR-induced increase in plasma [NO_2_^−^] has previously been reported to enhance performance in continuous sub-maximal endurance exercise, at least in recreationally active/moderately trained subjects (e.g., Bailey et al. 2015; Breese et al. 2013; Vanhatalo et al. 2011; Wylie et al. 2013a). However, it is important to note that the reduction of NO_2_^−^ to NO is enhanced as PO_2_ (Castello et al. 2006) and pH (Modin et al. 2001) decline, and recent reports suggest that the physiological and performance effects of NO_3_^−^ supplementation are enhanced in type II muscle compared to type I muscle or when type II fiber recruitment is expected to be greater (Bailey et al. 2015; Breese et al. 2013; Coggan et al. 2015; Ferguson et al. 2013, 2015; Hernández et al. 2012). Therefore, given that muscle PO_2_ and pH decline (Richardson et al. 1995) and the recruitment of type II muscle is greater (Essén 1978; Green 1978; Krustrup et al. 2004, 2009; Thomson et al. 1979) as exercise intensity increases, we reasoned that BR supplementation would be effective in enhancing performance during the intermittent high-intensity exercise protocols employed in this study.

Consistent with our experimental hypothesis and some previous reports of improved intermittent exercise performance after BR supplementation (Aucouturier et al. 2015; Bond et al. 2012; Thompson et al. 2015; Wylie et al. 2013a), performance (MPO) in the 24 × 6-s protocol was 5 % greater with BR relative to PL. When the 24 × 6-s protocol was divided into 25 % completion segments, MPO was significantly improved with BR in sprints 1–6 (+7 %), but not in sprints 7–12 (+4 %; *P* = 0.18), 13–18 (+5 %; *P* = 0.12) or 19–24 (+5 %; *P* = 0.14). These findings are consistent with recent observations that BR supplementation increased total work done during the first of two 40-min halves comprising repeated 2-min blocks of a 6-s all-out sprint, 100-s active recovery, and 20 s of rest (Thompson et al. 2015). However, in contrast to our experimental hypothesis, performance was not significantly impacted by BR supplementation in the 7 × 30-s or 6 × 60-s intermittent exercise protocols. Some previous studies have also reported no improvement in high-intensity intermittent exercise performance following BR supplementation (Christensen et al. 2013; Martin et al. 2014; Muggeridge et al. 2013). It should be acknowledged that, since subjects were asked to complete the three intermittent exercise protocols on consecutive days within each supplementation period, it is possible that basal fatigue increased across the series of tests and that this impacted performance during the second and/or third test days. However, since the participants were accustomed to high-intensity intermittent exercise, it is unlikely that changes in basal fatigue resistance would have substantially impacted our results.

Inconsistency in the efficacy of BR supplementation to improve high-intensity intermittent exercise performance in previous studies can be attributed, in part, to inter-study differences in participant training status, exercise modality, exercise protocol, and NO_3_^−^ supplementation procedures. Indeed, studies that have reported no improvement in intermittent exercise performance after BR supplementation have tested highly trained endurance athletes (Christensen et al. 2013; Muggeridge et al. 2013) and/or administered a low (<5 mmol NO_3_^−^) acute dose of BR (Martin et al. 2014; Muggeridge et al. 2013). In contrast, studies that have reported improved intermittent exercise performance following BR supplementation have involved recreationally active or moderately trained participants (Aucouturier et al. 2015; Thompson et al. 2015; Wylie et al. 2013b) and/or administered more chronic (≥3 days) BR supplementation (Aucouturier et al. 2015; Bond et al. 2012; Thompson et al. 2015) or a large (~29 mmol NO_3_^−^) dose of BR administered over 24 h (Wylie et al. 2013b). Another complication when interpreting the existing literature with regard to the efficacy of BR supplementation is that previous studies employed single intermittent exercise performance tests that have differed considerably with regard to work and rest intensities, work and rest durations, work-to-rest ratio, and number of work intervals (Aucouturier et al. 2015; Bond et al. 2012; Christensen et al. 2013; Martin et al. 2014; Muggeridge et al. 2013; Thompson et al. 2015; Wylie et al. 2013a). The findings of the present study therefore make an important contribution to our understanding of the effectiveness of BR supplementation to improve performance in different intermittent exercise protocols.

In contrast to the reduced steady-state $\dot{V}$O_2_ that has previously been observed during submaximal constant work rate exercise following nitrate supplementation (e.g. Bailey et al. 2009; Larsen et al. 2007; Wylie et al. 2013a; see Jones 2014 for review), $\dot{V}$O_2_ was not altered during any of the intermittent exercise protocols in the present study, which is consistent with our recent observations (Thompson et al. 2015). Therefore, the improved intermittent exercise performance in the 24 × 6-s protocol was not a function of changes in whole-body O_2_ consumption. Given reports that NO_3_^−^ supplementation is more effective at enhancing physiological responses and performance in type II compared to type I muscle (Ferguson et al. 2013, 2015; Hernández et al. 2012) or in situations where type II muscle fiber recruitment is expected to be greater (Bailey et al. 2015; Breese et al. 2013; Coggan et al. 2015), the differing effects of BR supplementation on performance in the different intermittent exercise protocols in the present study might be linked to differences in the muscle fiber recruitment patterns, and contribution to force production, across the different protocols tested. Studies assessing muscle fiber recruitment patterns from single-fiber high-energy phosphate depletion suggest that the recruitment of type II muscle is greater in 6 s sprints than 30 s sprints (Casey et al. 1996; Esbjörnsson-Liljedahl et al. 1999; Gray et al. 2008; Karatzaferi et al. 2001). The improved performance in the 24 × 6-s protocol, but not the 7 × 30-s or 6 × 60-s protocols, might therefore be linked to improved force production of type II muscle (Coggan et al. 2015; Hernández et al. 2012) as a consequence of increased sarcoplasmic reticulum calcium release (Hernández et al. 2012) and/or improved perfusion/oxygenation (Ferguson et al. 2013, 2015) in type II muscle. Increased perfusion of type II muscle would be particularly important given that the decline in muscle PCr is an important determinant of fatigue development during maximal-intensity intermittent exercise (Fulford et al. 2013; Gaitanos et al. 1993) and that muscle PCr resynthesis in recovery is an O_2_-dependent process (Trump et al. 1996; Vanhatalo et al. 2011). However, given that type II fibers are also heavily recruited during a 30-s all-out cycling sprint (Esbjörnsson-Liljedahl et al. 1999), it is unclear why dietary nitrate supplementation did not improve performance, at least during the first 30-s sprint, in the 7 × 30-s protocol.

A novel observation was that the increase in blood [lactate] from baseline to the end of exercise was greater after BR supplementation in the 24 × 6-s and 7 × 30-s protocols, although this was not the case in the 6 × 60-s protocol. Interestingly, RER was also significantly elevated after BR supplementation in the 24 × 6-s protocol. It is uncertain to what extent these changes contributed to enhanced performance with BR. In the 7 × 30-s protocol, performance was not enhanced despite the greater blood [lactate]; in the 24 × 6-s protocol, blood [lactate] was only greater with BR after sprints 22 and 24 where performance was not significantly enhanced (although it should be noted that there will be a temporal lag between muscle lactate production and the appearance of lactate in the blood; Jorfeldt et al. 1978). It has previously been reported that BR supplementation does not influence muscle pH during maximal-intensity intermittent exercise (Fulford et al. 2013) or alter glycolytic ATP turnover during continuous sub-maximal exercise (Bailey et al. 2010). Therefore, the increased blood lactate accumulation during the 24 × 6-s and 7 × 30-s protocols after BR supplementation may not necessarily reflect increased ATP flux through anaerobic glycolysis, but additional research is required before this possibility can be excluded. Instead, the increased blood lactate accumulation during these tests might be a function of increased perfusion of type II muscle following BR supplementation (Ferguson et al. 2013, 2015) since lactate production is greater in type II muscle (Esbjörnsson-Liljedahl et al. 1999) and lactate efflux can be increased with a greater muscle perfusion (Juel 1997). Further research is required to investigate the underlying mechanisms for the improved performance during short-duration maximal-intensity intermittent exercise following BR supplementation.

The present study provides important new insights into the efficacy of BR supplementation to improve performance in different types of intermittent exercise, and specifically indicates that BR supplementation enhanced performance during the 24 × 6-s protocol in which 6-s sprints were separated by 24 s of recovery. Repeated bouts of short-duration, high-intensity exercise interspersed with brief recovery intervals is a hallmark of many invasion games such as Association football, rugby union/league, and field hockey (King et al. 2009; Mohr et al. 2003; Spencer et al. 2004). It is interesting, therefore, that BR supplementation was most effective at enhancing performance in the intermittent exercise test that most closely resembled the exercise patterns manifest during many team sports, findings which are consistent with Thompson et al. (2015). Although performance was only significantly improved (by 7 %) after BR supplementation over the first six sprints of the 24 × 6-s protocol, it is possible that the non-significant 4–5 % increase in power output over the remaining 18 sprints might represent a practically meaningful improvement in performance in intermittent team sports. However, further research using validated field tests is required to assess the potential of dietary nitrate supplementation to improve team sport performance. Moreover, it should be acknowledged that a larger sample size than was used in the present study might be required to detect changes that are small but potentially practically meaningful in short-duration high-intensity intermittent exercise performance after BR supplementation.

In conclusion, the results of this study suggest that short-term BR supplementation, which increased the circulating plasma [NO_2_^−^], can improve performance during 24 6-s all-out sprints interspersed with 24 s of recovery. This improvement arose chiefly as a result of a specific performance enhancement over the first six sprints of this test protocol. However, despite a comparable increase in plasma [NO_2_^−^], BR supplementation did not significantly improve performance during seven 30-s all-out sprints interspersed with 4 min of recovery or six 60-s self-paced maximal efforts interspersed with 60 s of recovery. These findings elucidate the conditions under which BR supplementation may be ergogenic during high-intensity intermittent exercise and invite further mechanistic and practical exposition.

## Abbreviations

ANOVA: Analysis of variance
ATP: Adenosine triphosphate
BR: NO_2_^−^-rich beetroot juice
LSD: Least significant difference
MPO: Mean power output
MPO_mean_: Mean of mean power output
NO: Nitric oxide
NO_2_^−^: Nitrite
NO_3_^−^: Nitrate
NOS: Nitric oxide synthase
O_2_: Oxygen
PCr: Phosphocreatine
PL: NO_3_^−^-depleted beetroot juice
PO_2_: Partial pressure of oxygen
PPO: Peak power output
PPO_mean_: Mean of peak power output
Rpm: Revolutions per minute
$$ \dot{V} $$O_2_: Oxygen uptake
$\dot{V}$O_2peak_: Peak oxygen uptake
∆: Change

## References

[CR1] Aucouturier J, Boissière J, Pawlak-Chaouch M, Cuvelier G, Gamelin FX (2015). Effect of dietary nitrate supplementation on tolerance to supramaximal intensity intermittent exercise. Nitric Oxide.

[CR2] Bailey SJ, Winyard P, Vanhatalo A, Blackwell JR, Dimenna FJ, Wilkerson DP, Tarr J, Benjamin N, Jones AM (2009). Dietary nitrate supplementation reduces the O_2_ cost of low-intensity exercise and enhances tolerance to high-intensity exercise in humans. J Appl Physiol.

[CR3] Bailey SJ, Fulford J, Vanhatalo A, Winyard PG, Blackwell JR, DiMenna FJ, Wilkerson DP, Benjamin N, Jones AM (2010). Dietary nitrate supplementation enhances muscle contractile efficiency during knee-extensor exercise in humans. J Appl Physiol.

[CR4] Bailey SJ, Varnham RL, DiMenna FJ, Breese BC, Wylie LJ, Jones AM (2015). Inorganic nitrate supplementation improves muscle oxygenation, O_2_ uptake kinetics, and exercise tolerance at high but not low pedal rates. J Appl Physiol.

[CR5] Bogdanis GC, Nevill ME, Boobis LH, Lakomy HK, Nevill AM (1995). Recovery of power output and muscle metabolites following 30 s of maximal sprint cycling in man. J Physiol.

[CR6] Bogdanis GC, Nevill ME, Boobis LH, Lakomy HK (1996). Contribution of phosphocreatine and aerobic metabolism to energy supply during repeated sprint exercise. J Appl Physiol.

[CR7] Bond H, Morton L, Braakhuis AJ (2012). Dietary nitrate supplementation improves rowing performance in well-trained rowers. Int J Sport Nutr Exerc Metab.

[CR8] Breese BC, McNarry MA, Marwood S, Blackwell JR, Bailey SJ, Jones AM (2013). Beetroot juice supplementation speeds O_2_ uptake kinetics and improves exercise tolerance during severe-intensity exercise initiated from an elevated metabolic rate. Am J Physiol Regul Integr Comp Physiol.

[CR9] Casey A, Constantin-Teodosiu D, Howell S, Hultman E, Greenhaff PL (1996). Metabolic response of type I and II muscle fibers during repeated bouts of maximal exercise in humans. Am J Physiol.

[CR10] Castello PR, David PS, McClure T, Crook Z, Poyton RO (2006). Mitochondrial cytochrome oxidase produces nitric oxide under hypoxic conditions: implications for oxygen sensing and hypoxic signaling in eukaryotes. Cell Metab.

[CR11] Cermak NM, Gibala MJ, van Loon LJ (2012). Nitrate supplementation’s improvement of 10-km time-trial performance in trained cyclists. Int J Sport Nutr Exerc Metab.

[CR12] Chidnok W, DiMenna FJ, Fulford J, Bailey SJ, Skiba PF, Vanhatalo A, Jones AM (2013). Muscle metabolic responses during high-intensity intermittent exercise measured by ^31^P-MRS: relationship to the critical power concept. Am J Physiol Regul Integr Comp Physiol.

[CR13] Christensen PM, Nyberg M, Bangsbo J (2013). Influence of nitrate supplementation on VO_2_ kinetics and endurance of elite cyclists. Scand J Med Sci Sports.

[CR14] Coggan AR, Leibowitz JL, Kadkhodayan A, Thomas DP, Ramamurthy S, Spearie CA, Waller S, Farmer M, Peterson LR (2015). Effect of acute dietary nitrate intake on maximal knee extensor speed and power in healthy men and women. Nitric Oxide.

[CR15] Esbjörnsson-Liljedahl M, Sundberg CJ, Norman B, Jansson E (1999). Metabolic response in type I and type II muscle fibers during a 30-s cycle sprint in men and women. J Appl Physiol.

[CR16] Essén B (1978). Glycogen depletion of different fibre types in human skeletal muscle during intermittent and continuous exercise. Acta Physiol Scand.

[CR17] Ferguson SK, Hirai DM, Copp SW, Holdsworth CT, Allen JD, Jones AM, Musch TI, Poole DC (2013). Impact of dietary nitrate supplementation via beetroot juice on exercising muscle vascular control in rats. J Physiol.

[CR18] Ferguson SK, Holdsworth CT, Wright JL, Fees AJ, Allen JD, Jones AM, Musch TI, Poole DC (2015). Microvascular oxygen pressures in muscles comprised of different fiber types: impact of dietary nitrate supplementation. Nitric Oxide.

[CR19] Fitzsimons M, Dawson B, Ware D, Wilkinson A (1993). Cycling and running tests of repeated sprint ability. Aust J Sci Med Sport.

[CR20] Fulford J, Winyard PG, Vanhatalo A, Bailey SJ, Blackwell JR, Jones AM (2013). Influence of dietary nitrate supplementation on human skeletal muscle metabolism and force production during maximum voluntary contractions. Pflugers Arch.

[CR21] Gaitanos GC, Williams C, Boobis LH, Brooks S (1993). Human muscle metabolism during intermittent maximal exercise. J Appl Physiol.

[CR22] Govoni M, Jansson EA, Weitzberg E, Lundberg JO (2008). The increase in plasma nitrite after a dietary nitrate load is markedly attenuated by an antibacterial mouthwash. Nitric Oxide.

[CR23] Gray SR, Söderlund K, Ferguson RA (2008). ATP and phosphocreatine utilization in single human muscle fibres during the development of maximal power output at elevated muscle temperatures. J Sports Sci.

[CR24] Green HJ (1978). Glycogen depletion patterns during continuous and intermittent ice skating. Med Sci Sports.

[CR25] Haider G, Folland JP (2014). Nitrate supplementation enhances the contractile properties of human skeletal muscle. Med Sci Sports Exerc.

[CR26] Hernández A, Schiffer TA, Ivarsson N, Cheng AJ, Bruton JD, Lundberg JO, Weitzberg E, Westerblad H (2012). Dietary nitrate increases tetanic [Ca^2+^]i and contractile force in mouse fast-twitch muscle. J Physiol.

[CR27] Jones AM (2014). Dietary nitrate supplementation and exercise performance. Sports Med.

[CR28] Jorfeldt L, Juhlin-Dannfelt A, Karlsson J (1978). Lactate release in relation to tissue lactate in human skeletal muscle during exercise. J Appl Physiol Respir Environ Exerc Physiol.

[CR29] Juel C (1997). Lactate-proton cotransport in skeletal muscle. Physiol Rev.

[CR30] Karatzaferi C, de Haan A, Ferguson RA, van Mechelen W, Sargeant AJ (2001). Phosphocreatine and ATP content in human single muscle fibres before and after maximum dynamic exercise. Pflugers Arch.

[CR31] Kelly J, Vanhatalo A, Bailey SJ, Wylie LJ, Tucker C, List S, Winyard PG, Jones AM (2014). Dietary nitrate supplementation: effects on plasma nitrite and pulmonary O_2_ uptake dynamics during exercise in hypoxia and normoxia. Am J Physiol Regul Integr Comp Physiol.

[CR32] King T, Jenkins D, Gabbett T (2009). A time-motion analysis of professional rugby league match-play. J Sports Sci.

[CR33] Krustrup P, Söderlund K, Mohr M, Bangsbo J (2004). The slow component of oxygen uptake during intense, sub-maximal exercise in man is associated with additional fibre recruitment. Pflugers Arch.

[CR34] Krustrup P, Söderlund K, Relu MU, Ferguson RA, Bangsbo J (2009). Heterogeneous recruitment of quadriceps muscle portions and fibre types during moderate intensity knee-extensor exercise: effect of thigh occlusion. Scand J Med Sci Sports.

[CR35] Larsen FJ, Ekblom B, Sahlin K, Lundberg JO, Weitzberg E (2007). Effects of dietary nitrate on oxygen cost during exercise. Acta Physiol.

[CR36] Lundberg JO, Weitzberg E (2009). NO generation from inorganic nitrate and nitrite: role in physiology, nutrition and therapeutics. Arch Pharm Res.

[CR37] Martin K, Smee D, Thompson KG, Rattray B (2014). No improvement of repeated-sprint performance with dietary nitrate. Int J Sports Physiol Perform.

[CR38] McCartney N, Obminski G, Heigenhauser GJ (1985). Torque-velocity relationship in isokinetic cycling exercise. J Appl Physiol.

[CR39] Mendez-Villanueva A, Edge J, Suriano R, Hamer P, Bishop D (2012). The recovery of repeated-sprint exercise is associated with PCr resynthesis, while muscle pH and EMG amplitude remain depressed. PLoS One.

[CR40] Modin A, Björne H, Herulf M, Alving K, Weitzberg E, Lundberg JO (2001). Nitrite-derived nitric oxide: a possible mediator of ‘acidic–metabolic’ vasodilation. Acta Physiol Scand.

[CR41] Mohr M, Krustrup P, Bangsbo J (2003). Match performance of high-standard soccer players with special reference to development of fatigue. J Sports Sci.

[CR42] Muggeridge DJ, Howe CCF, Spendiff O, Pedlar C, James PE, Easton C (2013). The effects of a single dose of concentrated beetroot juice on performance in trained flatwater kayakers. Int J Sport Nutr Exerc Metab.

[CR43] Porcelli S, Ramaglia M, Bellistri G, Pavei G, Pugliese L, Montorsi M, Rasica L, Marzorati M (2015). Aerobic fitness affects the exercise performance responses to nitrate supplementation. Med Sci Sports Exerc.

[CR44] Richardson RS, Noyszewski EA, Kendrick KF, Leigh JS, Wagner PD (1995). Myoglobin O_2_ desaturation during exercise. Evidence of limited O_2_ transport. J Clin Invest.

[CR45] Spencer M, Lawrence S, Rechichi C, Bishop D, Dawson B, Goodman C (2004). Time-motion analysis of elite field hockey, with special reference to repeated-sprint activity. J Sports Sci.

[CR46] Stamler JS, Meissner G (2001). Physiology of nitric oxide in skeletal muscle. Physiol Rev.

[CR47] Thompson C, Wylie LJ, Fulford J, Kelly J, Black MI, McDonagh ST, Jeukendrup AE, Vanhatalo A, Jones AM (2015). Dietary nitrate improves sprint performance and cognitive function during prolonged intermittent exercise. Eur J Appl Physiol.

[CR48] Thomson JA, Green HJ, Houston ME (1979). Muscle glycogen depletion patterns in fast twitch fibre subgroups of man during submaximal and supramaximal exercise. Pflugers Arch.

[CR49] Trump ME, Heigenhauser GJ, Putman CT, Spriet LL (1996). Importance of muscle phosphocreatine during intermittent maximal cycling. J Appl Physiol.

[CR50] Vanhatalo A, Fulford J, Bailey SJ, Blackwell JR, Winyard PG, Jones AM (2011). Dietary nitrate reduces muscle metabolic perturbation and improves exercise tolerance in hypoxia. J Physiol.

[CR51] Waldron M, Highton J (2014). Fatigue and pacing in high-intensity intermittent team sport: an update. Sports Med.

[CR52] Wylie LJ, Kelly J, Bailey SJ, Blackwell JR, Skiba PF, Winyard PG, Jeukendrup AE, Vanhatalo A, Jones AM (2013). Beetroot juice and exercise: pharmacodynamic and dose-response relationships. J Appl Physiol.

[CR53] Wylie LJ, Mohr M, Krustrup P, Jackman SR, Ermιdis G, Kelly J, Black MI, Bailey SJ, Vanhatalo A, Jones AM (2013). Dietary nitrate supplementation improves team sport-specific intense intermittent exercise performance. Eur J Appl Physiol.

